# Monkeypox 2022: A Primer and Identify-Isolate-Inform (3I) Tool for Emergency Medical Services Professionals

**DOI:** 10.1017/S1049023X22001121

**Published:** 2022-10

**Authors:** Kristi L. Koenig, Christian K. Beÿ, Aileen M. Marty

**Affiliations:** 1. County of San Diego, Emergency Medical Services Office, Public Safety Group – San Diego County Fire, San Diego, California, USA; 2. Department of Emergency Medicine and Public Health, University of California Irvine, Orange, California, USA; 3. College of Arts, Sciences, and Education and Department of Translational Medicine, Herbert Wertheim College of Medicine, Florida International University, Miami, Florida, USA

**Keywords:** emergency medical dispatch, Emergency Medical Services, infectious disease outbreak, monkeypox, patient isolation

## Abstract

Monkeypox 2022 exhibits unprecedented human-to-human transmission and presents with different clinical features than those observed in prior outbreaks. Previously endemic only to West and Central Africa, the monkeypox virus spread rapidly world-wide following confirmation of a case in the United Kingdom on May 7, 2022 of an individual that had traveled to Nigeria. Detection of cases with no travel history confirms on-going community spread. Emergency Medical Services (EMS) professionals will likely encounter patients suspected or confirmed to have monkeypox, previously a rare disease and therefore unfamiliar to most clinicians. Consequently, it is critical for EMS medical directors to immediately implement policies and procedures for EMS teams – including emergency medical dispatchers – to identify potential monkeypox cases. These must include direction on actions EMS professionals should take to protect themselves and others from virus transmission. Monkeypox 2022 may manifest more subtly than it has historically. Presentations include a subclinical prodrome and less dramatic skin lesions – potentially limited to genital or anal body regions – which can be easily confused with dermatologic manifestations of common sexually transmitted infections (STIs). While most readily spread by close contact with infectious skin lesions on a patient, it is also transmissible from fomites, such as bed sheets. Additionally, droplet transmission can occur, and the virus can be spread by aerosolization under certain conditions. The long incubation period could have profound negative consequences on EMS staffing if clinicians are exposed to monkeypox. This report summarizes crucial information needed for EMS professionals to understand and manage the monkeypox 2022 outbreak. It presents an innovative Identify-Isolate-Inform (3I) Tool for use by EMS policymakers, educators, and clinicians on the frontlines who may encounter monkeypox patients. Patients are identified as potentially exposed or infected after an initial assessment of risk factors with associated signs and symptoms. Prehospital workers must immediately don personal protective equipment (PPE) and isolate infectious patients. Also, EMS professionals must report exposures to their agency infection control officer and alert health authorities for non-transported patients. Prehospital professionals play a crucial role in emerging and re-emerging infectious disease mitigation. The monkeypox 2022 3I Tool includes knowledge essential for all clinicians, plus specific information to guide critical actions in the prehospital environment.

## Introduction

On July 23, 2022, the World Health Organization (WHO; Geneva, Switzerland) declared the monkeypox 2022 outbreak the seventh-ever Public Health Emergency of International Concern (PHEIC). Monkeypox 2022 is a viral illness whose transmission rate and clinical features have shifted, enabling it to produce an unprecedented, rapidly expanding outbreak in non-endemic regions world-wide.^1^ Sustained human-to-human and community transmission are well-documented.^2^ Phylogenetic data indicate that the monkeypox virus in the 2022 outbreak has acquired significant mutations,^3^ which may account for its increased human-to-human transmission and altered clinical features. Human monkeypox has likely been circulating as a cross-continent, cryptic human transmission for some time;^4^ however, the first 2022 case outside of Africa was confirmed in the United Kingdom on May 7th in an individual with a history of travel to Nigeria.^5^ Case numbers precipitously increased in subsequent days, and epidemiological data revealed that many cases were not associated with travel to endemic regions.

As of July 25, 2022, confirmed cases rose to 17,156 in at least 69 non-endemic countries.^6^ Patients in the 2022 outbreak frequently present with non-classical signs and symptoms. While monkeypox may not be as easily transmissible as coronavirus disease 2019 (COVID-19), the escalating outbreak is a public health emergency and can lead to reverse zoonosis with the creation of new endemic areas, recurrent outbreaks, and negative effects on health care staffing. In addition, if containment measures are not immediately implemented, there is a high risk of the virus swiftly moving into vulnerable populations, such as children, pregnant women, the elderly, and the immunocompromised, leading to higher morbidity and mortality. As of July 2022, limited testing capacity and vaccine supply contribute to this concern.

**Figure 1.**
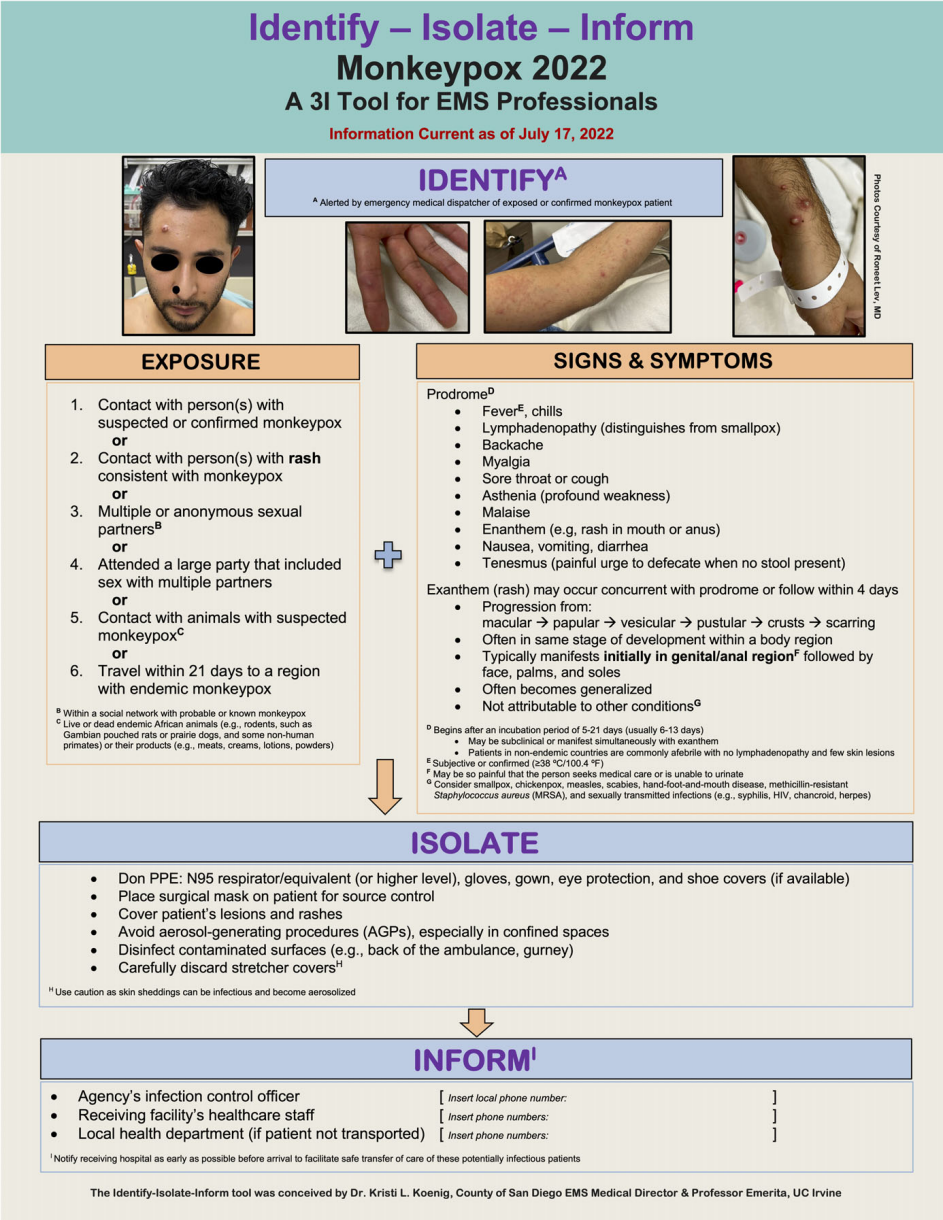
Monkeypox 2022 Identify-Isolate-Inform (3I) Tool for EMS Professionals. Abbreviations: 3I, Identify-Isolate-Inform; EMS, Emergency Medical Services; PPE, personal protective equipment.

This research report provides a concise summary of current knowledge about monkeypox and a novel Identify-Isolate-Inform (3I) Tool (Figure 1) for use by Emergency Medical Services (EMS) medical directors, policymakers, educators, and frontline EMS professionals who may encounter a suspected or confirmed case. The Appendix (available online only) contains a fillable version of the 3I Tool that can be customized with local contact information.

## Background

### Historical Monkeypox Clinical Presentation

Classically, monkeypox patients present with signs and symptoms resembling smallpox, a disease declared “globally eradicated” by the WHO in 1980.^7^ The incubation period ranges from five to 21 days (typically between six to 13 days). The first signs and symptoms are constitutional and called a prodrome because they manifest before the rash. These include influenza-like illness (fever, chills, sore throat, cough, malaise, asthenia, diarrhea, nausea, and vomiting);^8^ lymphadenopathy (a distinguishing feature from smallpox); and enanthem, often in the mouth and anus. Prodromal signs and symptoms typically precede the exanthem (rash) by one to four days. While the route of infection can influence clinical manifestations,^8^ initial skin lesions in human^9^ and non-human primates^10^ often present in the anogenital region and then appear on the face, palms, and soles before spreading centrifugally. Skin lesions progress through macular, papular, vesicular (blister), and pustular stages, followed by scabbing and healing. In addition, lesions present in the same stage of development in a given body region. Long-term sequelae of monkeypox infection can include disfiguring scars^11^ and permanent blindness resulting from corneal lesions.^12^

### 2022 Monkeypox Clinical Presentation

**Figure 2.**
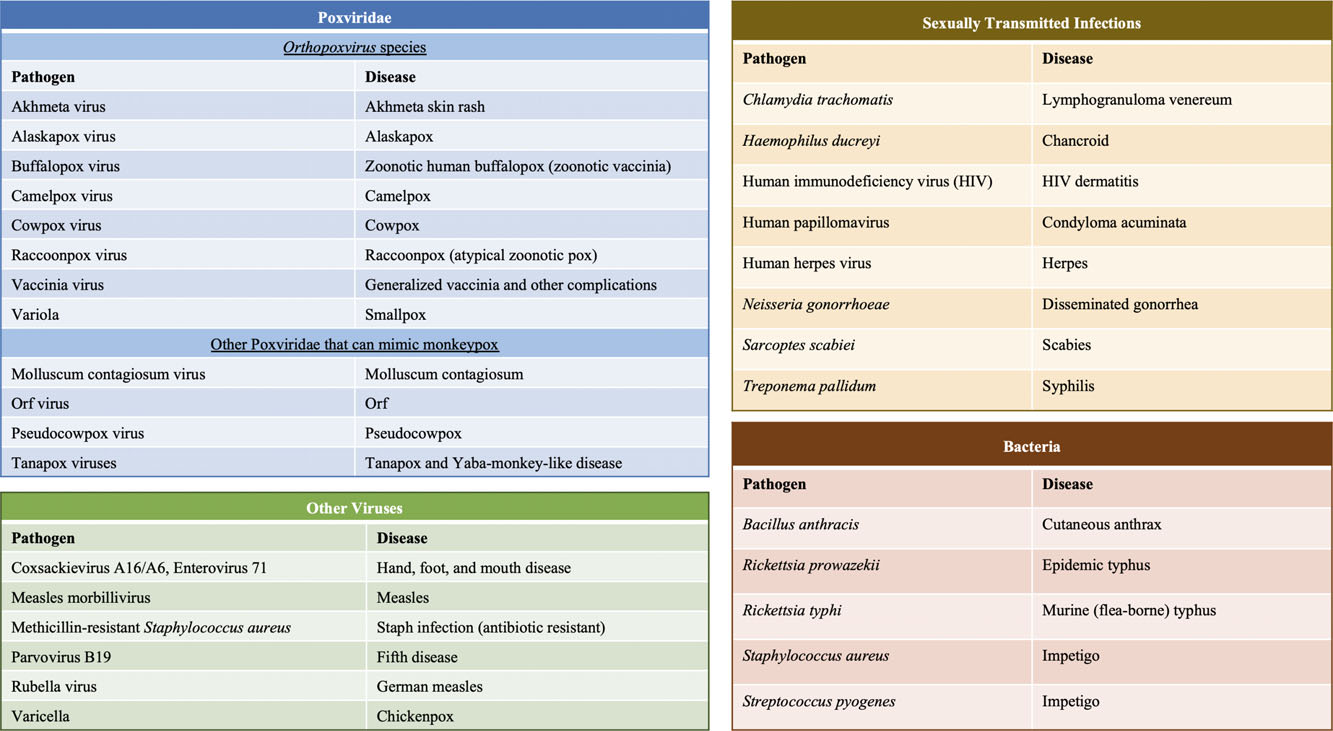
Selected Agents in the Differential Diagnosis of Monkeypox.

Unlike the classical monkeypox presentation, cases from the 2022 outbreak may not begin with a prodrome, skin lesions tend to be subdued, and lesions in a given body region may be at various stages of development. Furthermore, the constitutional symptoms can manifest simultaneously with the rash and enanthem. Moreover, lymphadenopathy and lesions on the palms and soles are less common. Hence, these features may have limited utility for differentiating monkeypox from other diseases. Monkeypox can appear similar to numerous more common diseases. The differential diagnosis of monkeypox is broad (Figure 2); thus, prehospital clinicians should have a low threshold for implementing monkeypox precautions.

Managing exposed persons in the 2022 outbreak can be challenging as the presentation may be subtle. Prehospital clinicians should consider monkeypox as a potential diagnosis in patients with risk factors for exposure because it can be infectious during a subclinical prodromal phase or before rash onset. Reducing monkeypox transmission helps avoid additional global stresses on the health care system. Strategies for reducing transmission include physical distancing, symptom monitoring, and standard hygienic practices. These approaches may obviate the need for quarantine of exposed health care workers, thereby contributing to preserving health care system capacity.^13^

**Figure 3.**
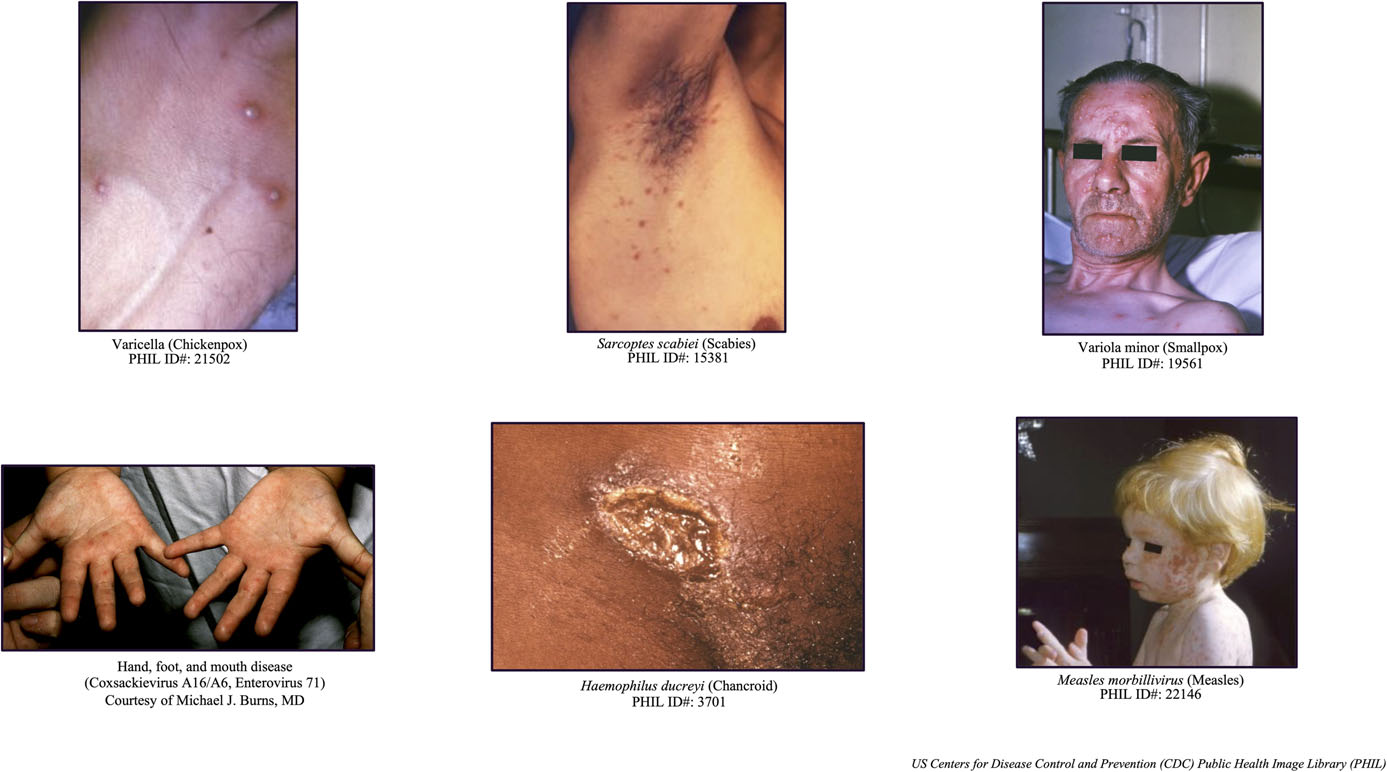
Selected Pathogens (Agents) in Monkeypox Differential Diagnosis.

Patients seeking care may exhibit only prodromal symptoms or be unaware of their infectious skin lesions. The differential diagnosis varies based on vaccination status, prior exposure, virus clade, and disease stage. Monkeypox can resemble various dermatological conditions and sexually transmitted infections (STIs; Figure 3).^14^ Distinguishing between monkeypox and other more common diseases in the prehospital setting can be challenging. Detecting alternate infections, especially other STIs, does not exclude the possibility of monkeypox. Hospitalization for monkeypox is rare and usually results from the need to treat secondary infections or manage intractable pain emanating from lesions.

Monkeypox virus is predominantly spread through close contact with infected skin lesions and can also be transmitted via bodily fluids and large droplets. Aerosolization of the virus (eg, from intubation, other medical procedures, or shaking contaminated bedsheets) can result in transmission to health care workers and others. Persons with confirmed monkeypox can mitigate transmission risk by covering infectious lesions, wearing a mask, and isolating themselves during the contagious period. Once lesions have healed, which can take two to four weeks or longer, they cease to be a source of infection.

## Report

Prehospital workers may be the first health care professionals to encounter suspected or confirmed monkeypox patients. Clinicians must learn to recognize the risk factors and presentations and consider monkeypox as a possible diagnosis. They should also take special precautions to prevent disease transmission and ensure the effective transfer of care at the receiving facility. More comprehensive data on the history, background, presentation, and management of monkeypox 2022 and the 3I concept are available.^14^

### Identify-Isolate-Inform (3I) Tool

The 3I framework was conceived in 2014 during the 2013-2016 Ebola virus outbreak when several Ebola cases presented at hospitals in the United States. The framework is designed to give clinicians high-level, actionable guidance for properly managing infectious diseases and has been applied to at least ten other conditions.^15–23^ Figure 1 presents a prehospital adaptation of the Monkeypox 3I Tool^14^ with specific considerations for EMS professionals.

### Identify

Work is on-going to ascertain whether suspected or confirmed monkeypox patients can be identified at the emergency medical dispatch level. While travel history remains important in determining disease exposure risk, documented community spread in non-endemic regions has limited its usefulness. After emergency medical dispatchers identify a person with suspected or confirmed monkeypox, EMS professionals are advised to implement appropriate infection prevention measures before patient contact. These are similar to those applied during the COVID-19 pandemic and may include:

- Requesting the patient to mask and come to the door of the residence, bringing medications, if possible (allowing first responders to remain outside);
- If first responders must go inside to evaluate a patient, first send in a scout paramedic wearing full personal protective equipment (PPE);
- Apply a surgical mask to the patient for source control (if tolerated); and
- Avoid aerosol-generating procedures (AGPs).

**Figure 4.**
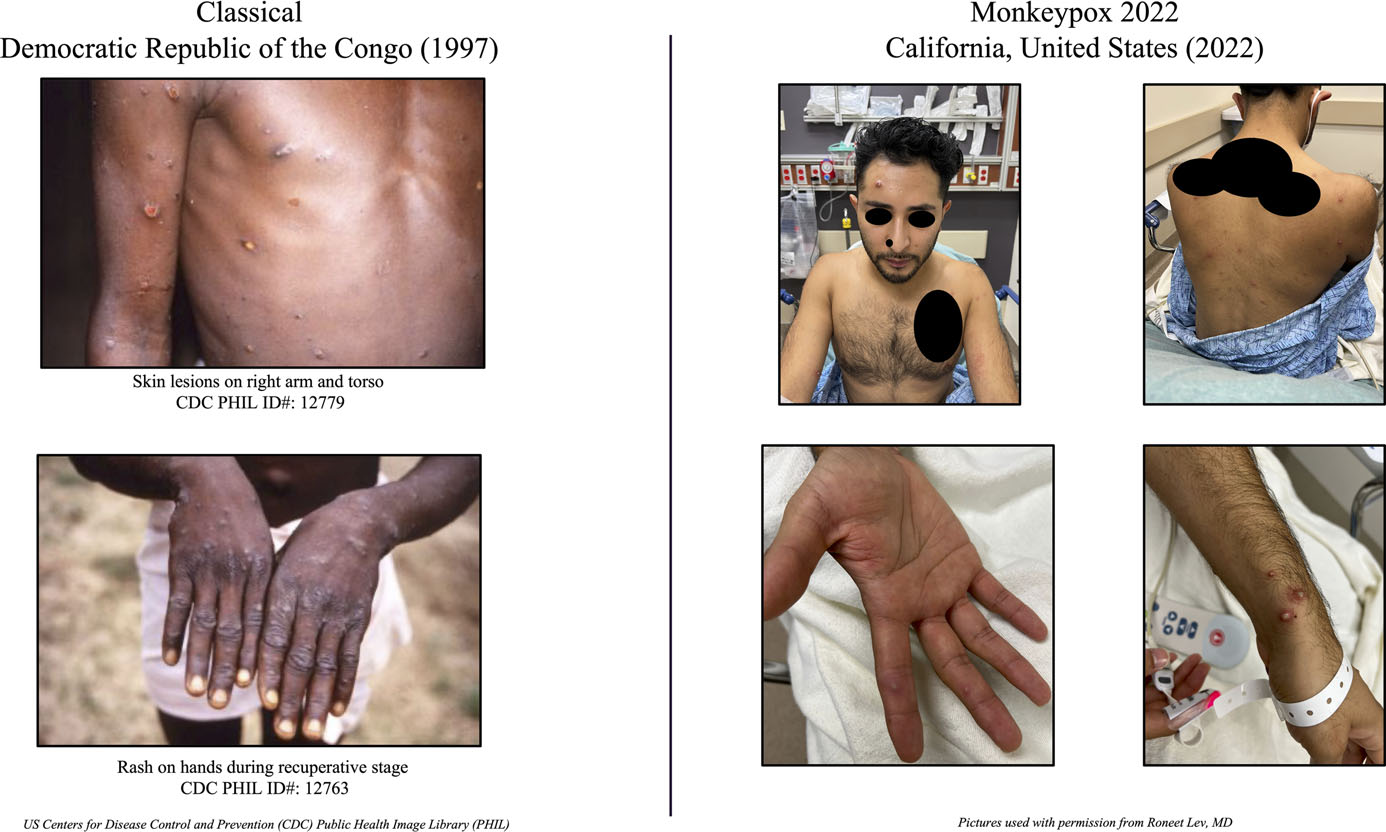
Classical Versus Monkeypox 2022 Presentations.

If monkeypox is suspected, prehospital professionals should determine the patient’s likelihood of having the disease based on signs and symptoms and exposure risk. Cases in the 2022 outbreak are less likely to present with classical clinical indicators (Figure 4). Exposure risks independent of travel history include contact with persons or animal reservoirs suspected or confirmed to have monkeypox and contact with infected surfaces or other formites.

### Isolate

Prehospital clinicians should don appropriate PPE throughout first contact, transport, care transfer, and during disinfection of the transport vehicle. Proper PPE includes an N95 respirator/equivalent (or higher), gloves, gown, eye protection,^24^ and shoe covers, if available. Notably, PPE used during the COVID-19 pandemic is sufficient to protect prehospital professionals from monkeypox. Source-control measures, including placing a surgical mask on the patient and covering exposed lesions, can lessen the risk of transmission in enclosed spaces (eg, the back of an ambulance). Similarly, avoid AGPs whenever possible because of the increased exposure risk and potential for infectious monkeypox virus aerosols to linger for extended periods in enclosed spaces.^25^

### Inform

It is imperative that transporting crews relay the patient’s suspected or confirmed monkeypox status to the receiving facility early to allow time for the hospital to prepare and minimize risks of infection spread and delays in the transfer of care. Local or state protocols may require prehospital clinicians to report communicable infectious diseases to their agency’s infection-control officer or health authority, especially for patients not transported to a hospital. Follow required protocols when reporting communicable diseases and health care worker exposures.

## Discussion

This research report discusses considerations for the management of suspected or confirmed monkeypox 2022 in the prehospital setting. As of July 11, 2022, a literature search of the PubMed (National Center for Biotechnology Information, National Institutes of Health; Bethesda, Maryland USA); MEDLINE (US National Library of Medicine, National Institutes of Health; Bethesda, Maryland USA); CINAHL (EBSCO Information Services; Ipswich, Massachusetts USA); and Scopus (Elsevier; Amsterdam, Netherlands) databases did not identify any published articles specifically addressing EMS considerations for monkeypox. Given the rapidly evolving nature of the 2022 outbreak, only limited data are available describing the clinical presentation, management, epidemiology, and long-term outcomes. Furthermore, the diagnosis cannot be confirmed in the prehospital setting. Despite this, applying the Monkeypox 3I Tool for EMS Professionals (Figure 1) can increase opportunities to detect presumptive monkeypox and reduce the risk of disease transmission.

As demonstrated by the COVID-19 pandemic, prehospital clinicians play a critical role in the management of public health emergencies. Throughout the pandemic, prehospital professionals have assisted with out-of-hospital treatments, vaccination campaigns, and patient transport, among other activities. Although monkeypox 2022 is unlikely to cause a global pandemic on the scale of COVID-19, it is nevertheless a major international public health threat. Prehospital clinicians can apply their unique training and role as the interface between at-risk patient populations and health care to mitigate the further spread of monkeypox 2022.

## Conclusion

Monkeypox 2022 represents a rapidly escalating outbreak with extensive and sustained global human-to-human and community transmission. Cases in the 2022 outbreak differ from classical clinical presentations as described in endemic regions of Central and West Africa and during the 2003 zoonotic monkeypox outbreak in the United States.^8^ Importantly, before this outbreak, monkeypox was very rare; thus, most health care workers have never encountered the disease. Expansion of cases to non-endemic regions creates a risk of reverse zoonosis and the creation of new endemic zones across the globe.

Prehospital clinicians play a key role in infectious disease mitigation, as demonstrated by the COVID-19 pandemic. The science on the monkeypox 2022 outbreak continues to evolve. It is imperative that clinicians, particularly those in the prehospital setting who may encounter suspected or confirmed cases, immediately *identify* and *isolate* such patients to protect themselves and other health care workers from exposure and *inform* the relevant health authorities to help mitigate the spread of this re-emerging infectious disease.

## Supplementary material

For supplementary material accompanying this paper visit https://doi.org/10.1017/S1049023X22001121. click here to view supplementary material
