# Supplementary material for: Monkeypox 2022: A Primer and Identify-Isolate-Inform (3I) Tool for Emergency Medical Services Professionals
Source: Prehosp Disaster Med. 2022 Aug 4;37(5):687–92. doi: 10.1017/S1049023X22001121 (PMC9470524; doi:10.1017/S1049023X22001121)
Supplement: Supplementary file 1 [file S1049023X22001121sup001.pdf]

Identify – Isolate – Inform

Monkeypox 2022

A 3I Tool for EMS Professionals

Information Current as of July 17, 2022

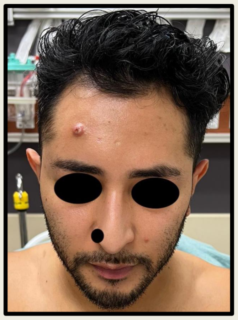

IDENTIFY[A]

[A] Alerted by emergency medical dispatcher of exposed or confirmed monkeypox patient

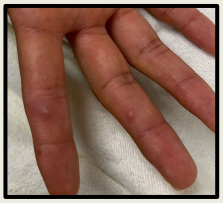

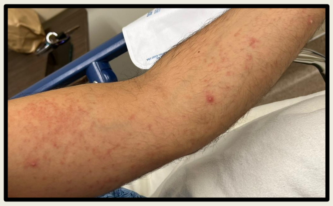

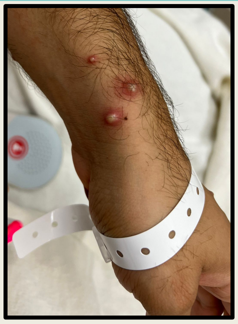

Photos Courtesy of Roneet Lev, MD

EXPOSURE

1. Contact with person(s) with suspected or confirmed monkeypox
   or
2. Contact with person(s) with **rash** consistent with monkeypox
   or
3. Multiple or anonymous sexual partners[B]
   or
4. Attended a large party that included sex with multiple partners
   or
5. Contact with animals with suspected monkeypox[C]
   or
6. Travel within 21 days to a region with endemic monkeypox

[B] Within a social network with probable or known monkeypox
[C] Live or dead endemic African animals (e.g., rodents, such as Gambian pouched rats or prairie dogs, and some non-human primates) or their products (e.g., meats, creams, lotions, powders)

+

SIGNS & SYMPTOMS

Prodrome[D]

- Fever[E], chills
- Lymphadenopathy (distinguishes from smallpox)
- Backache
- Myalgia
- Sore throat or cough
- Asthenia (profound weakness)
- Malaise
- Enanthem (e.g, rash in mouth or anus)
- Nausea, vomiting, diarrhea
- Tenesmus (painful urge to defecate when no stool present)

Exanthem (rash) may occur concurrent with prodrome or follow within 4 days

- Progression from:
  macular → papular → vesicular → pustular → crusts → scarring
- Often in same stage of development within a body region
- Typically manifests **initially in genital/anal region**[F] followed by face, palms, and soles
- Often becomes generalized
- Not attributable to other conditions[G]

[D] Begins after an incubation period of 5-21 days (usually 6-13 days)
- May be subclinical or manifest simultaneously with exanthem
- Patients in non-endemic countries are commonly afebrile with no lymphadenopathy and few skin lesions

[E] Subjective or confirmed (≥38 °C/100.4 °F)
[F] May be so painful that the person seeks medical care or is unable to urinate
[G] Consider smallpox, chickenpox, measles, scabies, hand-foot-and-mouth disease, methicillin-resistant *Staphylococcus aureus* (MRSA), and sexually transmitted infections (e.g., syphilis, HIV, chancroid, herpes)

ISOLATE

- Don PPE: N95 respirator/equivalent (or higher level), gloves, gown, eye protection, and shoe covers (if available)
- Place surgical mask on patient for source control
- Cover patient's lesions and rashes
- Avoid aerosol-generating procedures (AGPs), especially in confined spaces
- Disinfect contaminated surfaces (e.g., back of the ambulance, gurney)
- Carefully discard stretcher covers[H]

[H] Use caution as skin sheddings can be infectious and become aerosolized

INFORM[I]

- Agency's infection control officer [*Insert local phone number:*]
- Receiving facility's healthcare staff [*Insert phone numbers:*]
- Local health department (if patient not transported) [*Insert phone numbers:*]

[I] Notify receiving hospital as early as possible before arrival to facilitate safe transfer of care of these potentially infectious patients

The Identify-Isolate-Inform tool was conceived by Dr. Kristi L. Koenig, County of San Diego EMS Medical Director & Professor Emerita, UC Irvine
